# Effects of Educational Video on Pre-operative Anxiety in Children - A Randomized Controlled Trial

**DOI:** 10.3389/fped.2021.640236

**Published:** 2021-05-12

**Authors:** Valentina Härter, Claus Barkmann, Christian Wiessner, Martin Rupprecht, Konrad Reinshagen, Julian Trah

**Affiliations:** ^1^Department of Pediatric Surgery, University Medical Center Hamburg-Eppendorf, Hamburg, Germany; ^2^Department of Child and Adolescent Psychiatry, University Medical Center Hamburg-Eppendorf, Hamburg, Germany; ^3^Department of Medical Biometry and Epidemiology, University Medical Center Hamburg-Eppendorf, Hamburg, Germany; ^4^Department of Pediatric Orthopedics, Altona Children's Hospital, Hamburg, Germany; ^5^Department of Orthopedics, University Medical Center Hamburg-Eppendorf (UKE), Hamburg, Germany

**Keywords:** anxiety, children, surgery, patient information, audio-visual intervention, RCT

## Abstract

**Objective:** Audio-visual interventions have been used to provide relevant patient information to reduce pre-operative anxiety in children. The aim of the study was to investigate whether self-reported state anxiety in children could be reduced by presenting a child-friendly educational video on the day of surgery.

**Methods:** A prospective, single-blinded, two-armed, randomized controlled study was designed with three measurement time points including 90 children (6–17 years) and their parents. In the intervention group (IG), the children and their parents were shown a child-friendly video explaining the perioperative procedures that would be applied during the hospital stay, in addition to receiving standard information. In the control group (CG), children and parents received standard information provided by the nursing staff. The primary outcome was any change in the children's pre-operative state anxiety levels, as measured by the State-Trait Operation Anxiety Inventory (STOA). A secondary outcome was patient satisfaction regarding the received information.

**Results:** Anxiety was significantly reduced in both groups after receiving either the intervention plus standard information or the standard information only. No significant difference in anxiety reduction was observed between the IG and the CG. However, the children and parents in the IG reported fewer worries than those in the CG.

**Conclusion:** A child-friendly, educational video can be an additional tool for providing patient information and reducing pre-operative anxiety in children and their parents. Further studies should focus on the timing of the intervention and on age- and developmentally appropriate information formats and contents to address children's pre-operative anxiety.

**Clinical Trial Registration:** Patient Anxiety Reduction in Children by Using Simple Explanation Videos, ID: NCT0441377; www.clinicaltrials.gov, Data Sharing Statement: Deidentified individual participant data will not be made available.

## Introduction

The presence of pre-operative anxiety in children and their parents before surgical interventions is clinically relevant to perioperative care. Surgery and hospitalization are stressful situations (1), and anxiety can negatively impact perioperative care and the patient's behaviors and ability to adjust to the hospital setting (2).

Previous studies have shown that high pre-operative anxiety states have a negative impact on post-operative pain experience (3) and increase post-operative anxiety levels in children (4, 5).

Self-reported state and trait anxiety evaluations are often used to assess patients' anxiety before surgery (6). State anxiety can be defined as an emotional reaction in response to a perceived threat that occurs at a specific point in time and with varying intensity, comprised of both affective and cognitive components of the current emotional state. Trait anxiety describes general feelings of worries and discomfort (7).

The experience of parental pre-operative anxiety regarding their child's operation is also an important issue that can affect the perioperative care setting (8, 9). Parental anxiety has been associated with distress and problematic behaviors in the child during and after hospitalization. Furthermore, unanswered questions and missing information regarding the operation can increase parental anxiety (8).

Children and their parents wish to be informed regarding the surgical intervention, anesthesia, potential pain problems, and other procedures they may undergo during the hospitalization period (10–13). The adaptation of information to meet the needs of children should focus on optimizing the timing, format, and content of the information being presented (14).

Various non-pharmacological interventions have shown that the provision of specific patient information can reduce pre-operative anxiety in children before an elective surgical procedure (15, 16). Research has increasingly focused on exploring the effects of audio-visual (17) and technology-based (18) interventions, particularly digital intervention programs.

A previous study concluded that the information presented in videos should be short and precise (17). Kain et al. showed that providing procedural information is sufficient to allow children to cope with the expected stress associated with the surgical intervention (19). Although evidence-based studies are important for pediatric pre-operative preparation, to our knowledge, no trials have focused on the effects of using an educational video with a laying technique (20). In this educational video new scenes are wiped in or out with two hands explaining complex information in a short and simple manner about hospital and care standards.

The questions under research were as follows:

1. Can a child-friendly, educational video reduce children's pre-operative state anxiety before elective surgery compared with the standard information procedure?
2. Does the use of such a video increase the reported patient satisfaction of the children and parents compared with the standard information procedure?

We expected that self-reported, pre-operative, state anxiety of children would decrease after the video intervention in addition to receiving standard information compared with children who received only the standard information procedure. Furthermore, we expected an increase in patient satisfaction among children and their parents who were shown the video compared with those who received standard information.

## Methods

### Trial Design

**Figure 1:**
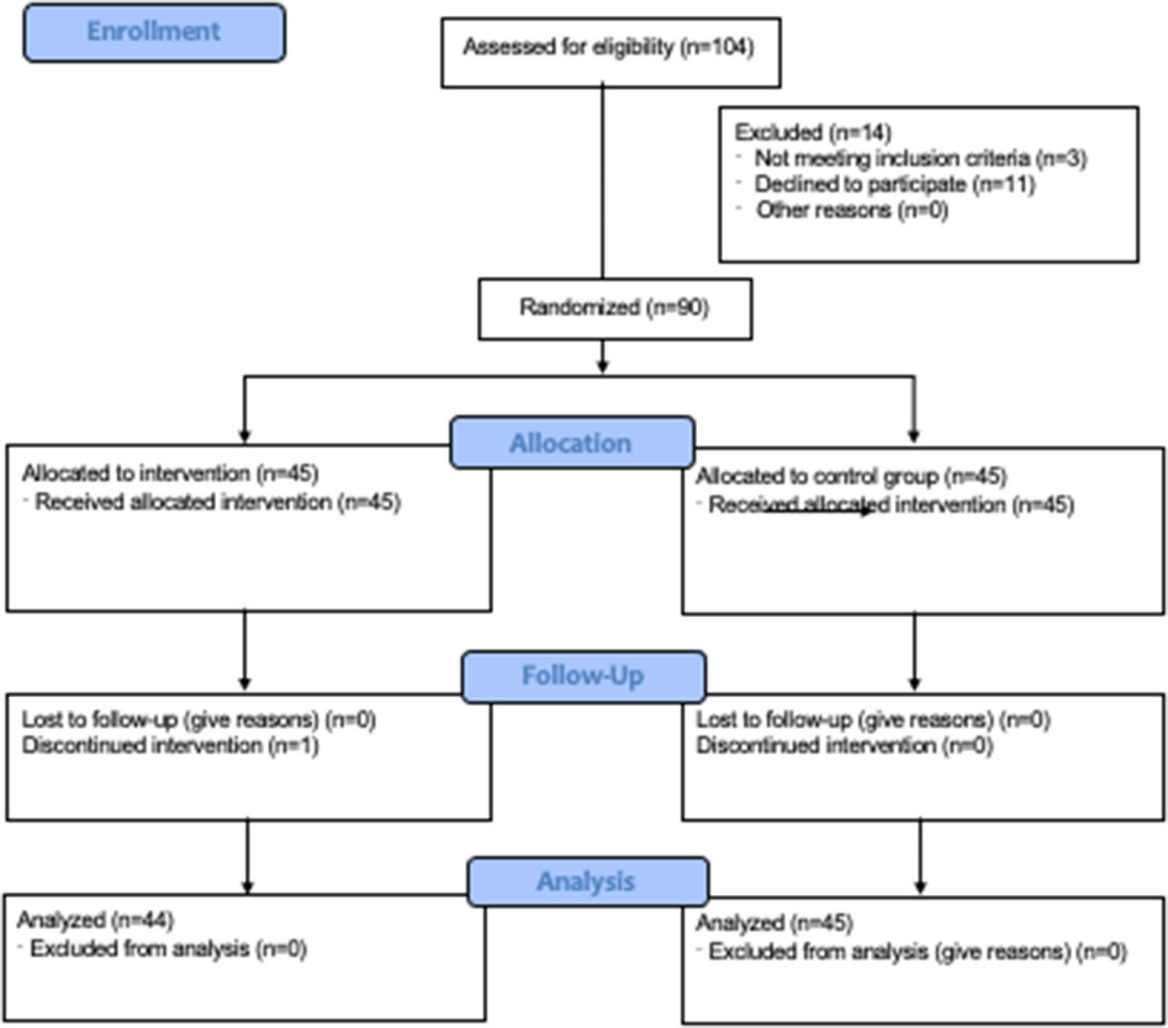
Consort 2010 flow diagram of the study.

The present study was conducted as a prospective intervention study with three measurement time points using a randomized-controlled design that included 90 participants. Data were collected from November 2019 until April 2020 at three primary care centers. The sources of information were children and their parents. Enrollment was conducted, as shown in Figure 1.

Families were randomized either to the intervention (IG, *n* = 45) or control arms (CG, *n* = 45). The children and parents included in the IG were shown a child-friendly, animated, educational video (see below), in addition to receiving standard information regarding the hospital stay. Standard information regarding the hospital stay was provided to all groups by the nursing staff.

Questionnaires were answered by both the child and one parent (mother or father) on the day of surgery, both before administering the intervention (t1) and directly after the intervention but before surgery (t2), and 1 day after surgery (t3).

### Participants

Children aged 6–17 years who were undergoing elective surgery were eligible for inclusion. All patients agreed to participate and were randomly allocated to one of the study groups (Figure 1). All participants received treatment as allocated. The surgical interventions were divided into body regions: chest, abdomen, extremities (including head and neck), anal region, or spine. Sufficient German language skills to understand the video were required for both the child and parents for inclusion.

Exclusion criteria included children with chronic illnesses, mental disorders, and the use of regular medication to avoid conflicting effects with regard to anxiety levels.

### Randomization

The randomization procedure was conducted as a written document (randomization table). Randomization was carried out on the day of the operation after assessing study eligibility by a member of the study team. Child and parent were informed that there were two groups, one of which they were randomly assigned. After being informed about the study, both received standard information procedure (TAU). The control group then completed the second questionnaire. The video group filled out the second questionnaire only after they had seen the educational video.

### Intervention

#### Intervention Group: Educational Video

On the day of surgery, before undergoing the standard information procedure [treatment as usual (TAU), see below], the IG participants and their parents were shown a short, educational video with animated laying technique. The animated laying technique is used in the educational video of this study. The technique is characterized by illustrations that is placed in or out of the screen by two hands. The illustrations show information in a simple and short way in <4 min. This simplicity should lead to complex information being conveyed in a playful, animated way and in visual language. This should help children to get access to the procedures that will expect them in their hospital stay. The video was presented on a handheld tablet in the patient, holding, or examination room. The video contained information regarding the procedures the child was likely to encounter during the hospital stay, which was presented in a child-friendly way. This information included the medical procedures that would occur before and after surgery and the procedures that would be encountered during post-operative care (see Appendix). After viewing the video, questions about the information contained in the video could be asked of a member of the study team, who was present during the entire video viewing process.

#### Control Group: Treatment-As-Usual

On the day of surgery, participants in the CG received the standard information procedure (TAU), which was provided by the nursing staff and varied in form, length, and timing. The information provided at each center was identical. If sufficient time was available, the patients could visit the surgical ward. The nursing staff explained the procedures before the operation, including the application of pre-medication and post-operative care.

### Outcomes and Instruments

#### Primary Outcome

The primary outcome of this study was the self-reported pre-operative state anxiety levels in children undergoing elective surgery as measured at t1 and t2 using the State-Trait Operation Anxiety Inventory (STOA) (2). The STOA is a psychometric questionnaire used to evaluate a patient's subjective fear of surgery. The questionnaire measures both state and trait anxiety. State anxiety can be further divided into cognitive and affective components using different subscales. Anxiety cognition is typically activated before the start of the operation, whereas the affective component increases strongly immediately before the stressful event, followed by a significant decrease (2). Parallel to the children's self-report, the parent self-report was assessed. For adults, the STOA has already been validated (2). For children and adolescents, psychometric analyses were conducted within this study (Cronbach's α, factor analysis), which confirm good reliability and factorial validity (e.g., α = 0.91 for children's and parents state anxiety at t1, manuscript in preparation).

#### Secondary Outcome

The secondary outcome measured was patient satisfaction with the received information, as rated by both the children and their parents. A study-specific questionnaire comprising eight items was adapted (21). It was used in former studies to evaluate patient satisfaction regarding the information obtained before a medical procedure. The selection was made on aspects of face validity and item content. We used one item (**Table 4**, item 3) from the usefulness scale of patient information material (USE) and two items from the scale's preliminary item pool (**Table 4**, item 4 and 5). The other items were developed according to the objectives of the study. The questionnaire hasn't been used in a similar patient population. It was given to the children and their parents in both groups the day after surgery (t3).

### Ethics

Approval was obtained from the Ethics Committee of the Hamburg Medical Association (Number PV6045). A written consent form was signed by study participants and their parents before study entry. The study was registered at https://clinicaltrials.gov/ under the ID: NCT04413773.

### Statistical Methods

Student's *t*-test and Chi-square-test were used to analyze sociodemographic data. The prediction of surgery anxiety was analyzed using a longitudinal, mixed model (22). A priori power calculations were based on the assumption that the study would detect 3-point differences for state anxiety scores between the IG and the CG after the intervention, resulting in a sample size of 45 participants for each group (80% power; α < 0.05, two-tailed). A *t*-test was used for independent samples to measure child and parent satisfaction. Missing data were treated using the Full Information Maximum Likelihood method (FIML). Statistical analysis was performed using SPSS Statistics 26.

## Results

### Participants

The demographic and clinical information of the participants is shown in Table 1. No significant differences were observed between the IG and the CG in terms of age, gender, education, language, clinic setting, the number of previous operations, the number or type of surgeries performed on the extremities or time.

**Table 1:** Sample description.

**Variable**	**Control group (*n* = 45)**	**Intervention group (*n* = 45)**	**Total sample (*n* = 90)**	**Test statistic**	***p***
Age in years *M* (SD)	12.63 (2.23)	12.37 (2.72)	12.50 (2.48)	*t* = −0.489	0.626
Gender *n* (%)				χ^2^ = 0.400	0.527
Female	21 (47)	24 (53)	45 (50)		
Male	24 (53)	21 (47)	45 (50)		
Education (*n*)				χ^2^ = 12.129	0.096
Elementary school	6	8	14		
Middle school	0	4	4		
High school	22	14	36		
Other	17	19	36		
Mother tongue German *n* (%)				χ^2^ = 2.000	0.157
No	5 (11)	10 (22)	15 (17)		
Yes	40 (89)	35 (78)	75 (83)		
Clinic *n* (%)				χ^2^ = 4.320	0.115
Primary care center 1	18 (40)	18 (40)	36 (40)		
Primary care center 2	23 (51)	27 (60)	50 (56)		
Primary care center 3	4 (9)	0 (0)	4 (4)		
Pre-operation *n* (%)				χ^2^ = 0.179	0.673
No	25 (56)	23 (51)	48 (53)		
Yes	20 (44)	22 (49)	42 (47)		
Surgery on extremities *n* (%)				χ^2^ = 1.538	0.215
No	5 (11)	10 (22)	15 (17)		
Yes	40 (89)	35 (78)	75 (83)		
Type of surgery (*n*)					
Minor	16	13	29		
Medium	25	24	49		
Major	3	8	11		
No data	1	0	1		

*The variable “Pre-operation” indicates if the child was previously operated or not*.

### Course of Anxiety Over Time

The descriptive distribution of operation-associated anxiety from t1 to t2 and t3 for both groups is demonstrated in Table 2. Children in both groups reported reduced state anxiety from t1 to t2, with anxiety scores shifting from a mean (M) of 10.5 [standard deviation (SD) = 7.12] to 9.6 (SD = 6.86), respectively, in the IG and from 9.4 (SD = 6.23) to 8.0 (SD = 6.46) in the CG.

**Table 2:** Distribution of surgery anxiety before (t1) and after (t2) intervention, and after surgery (t3).

			**CG**			**IG**		
			***n***	***M***	**SD**	***n***	***M***	**SD**
Self-report	STOA-State	t1	44	9.4	6.23	45	10.5	7.12
		t2	40	8.0	6.46	44	9.6	6.86
		t3	38	6.2	6.64	43	5.7	5.74
	STOA-Trait	t1	44	12.1	8.71	45	13.6	11.26
		t2	40	9.9	9.20	44	11.1	11.99
		t3	40	9.3	8.72	43	11.0	11.21
Parent report	STOA-State	t1	43	10.7	5.90	40	9.6	6.43
		t2	40	9.1	5.83	39	8.2	5.72
		t3	39	3.5	4.09	36	4.1	4.55
	STOA-Trait	t1	43	20.1	10.47	40	20.7	10.85
		t2	40	17.6	10.26	39	16.8	9.80
		t3	39	16.4	8.70	36	16.1	7.95

*State anxiety is assessed with 10 items. The resulting score has a range from 0 to 30 with higher values indicating higher state anxiety. Children and parents respond to a 4-point rating scale (0 = not at all, 1 = somewhat, 2 = moderately so, 3 = very much so, Cronbach's alpha = 0.90), which describes the intensity of their momentary experienced anxiety. Trait anxiety consists of 20 items. The resulting score has a range from 0 to 60 with higher values indicating higher trait anxiety. Under the instruction “When I think about operations and anesthesia in general, I worry that.” patients should indicate the frequency of occurrence of such fears based on a 4-point Likert scale (e.g., “.one can still feel something of the operation despite the anesthesia”; “.pain occurs after the operation”) on 0–3 (0 = almost never, 1 = sometimes, 2 = often, 3 = almost always, Cronbach's alpha = 0.92)*.

State anxiety decreased significantly compared with trait anxiety, especially at t3. Self- and parent-reported anxiety levels demonstrated similar but not identical patterns, with children rating state anxiety higher than trait anxiety.

Anxiety values tended to be in the lower range of the scale (slightly) and to decrease over time. Parents assessed their state and trait anxiety levels regarding their children's operations similarly but reported lower levels of state anxiety than trait anxiety after the operation.

In addition, no significant differences in the cognitive and affective components of state anxiety were observed between the IG and CG in children (not depicted).

### Effect of the Video

Table 3 shows the test of the hypothesis using the longitudinal, mixed model. The control variables that were considered included age, gender, native speaker, previous surgery, region of surgery and time (t1-3). The numbers represent the mean anxiety values for each respective STOA scale, showing a starting constant value and the changes associated with the predictor-specific components. In the longitudinal multilevel analysis, a separate regression equation for the dependent variable over time is calculated first for each individual case (level 1) (22). Intercepts are the initial values at t1, slopes are the rates of change per year. The intercepts and slopes of all cases can now in turn be taken as variables and analyzed *via* (level 2) regression equations again. The mean values of the intercepts and slopes are referred to as fixed effects or regression coefficients, respectively. As shown in the table, state anxiety, as measured using the self-reported STOA scale, can be optimally predicted by starting with a constant value of 3.2 anxiety points, plus the age of each participant multiplied by 0.6. Each predictor-specific component is associated with an additional adjustment, such as the reduction of 2.9 anxiety points for boys or the addition of 3.1 anxiety points for non-native speakers. The interaction between the difference in anxiety scores between time t1 and t2 and the group, as shown on the lower part of Table 3, indicated no significant effect on the change in self-reported pre-operative state anxiety in children between before and after the intervention for the IG compared with the CG (*M* = 0.3, SE = 0.74, not significant). However, significant reductions in both state and trait anxiety were observed for both groups between t1 and t2 and between t1 and t3 in both the patient- and parent-reported surveys.

**Table 3:** Prediction of surgery anxiety before (t1), after (t2) intervention, and after surgery (t3).

	**Self-report**		**Self-report**		**Parent report**		**Parent report**	
	**State-anxiety**		**Trait-anxiety**		**State-anxiety**		**Trait-anxiety**	
	**Effect**	**SE**	**Effect**	**SE**	**Effect**	**SE**	**Effect**	**SE**
**Fixed effects**								
Constant	3.23	3.65	−6.38	5.69	10.05	3.73	21.96	6.26
Age in years	0.57^*^	0.26	1.32^**^	0.41	−0.08	0.27	−0.25	0.45
Gender (boys)	−2.91^*^	1.30	−3.11	2.04	0.98	1.33	1.15	2.22
Native speaker (no)	3.09	1.76	6.28^*^	2.74	4.37^*^	1.75	4.65	2.94
Previous surgeries (yes)	−1.99	1.29	−0.85	2.02	−0.98	1.30	−2.31	2.15
Surgery region (extremities)	1.29	1.73	3.67	2.72	1.24	1.72	1.26	2.87
**Time**								
t1-t2	−1.30^*^	0.54	−2.14^***^	0.57	−1.24^***^	0.33	−2.15^**^	0.81
t1-t3	−3.06^**^	0.94	−2.24^*^	1.09	−6.74^***^	0.94	−3.38^*^	1.30
Group (video)	0.91	1.35	1.31	1.05	−1.18	0.33	0.55	2.34
**Time** **×** **group (video)**								
t1-t2 × group	0.29	0.74	−0.50	0.79	0.22	0.47	−1.66	1.15
t1-t3 × group	−1.95	1.30	−0.68	1.51	1.81	1.36	−0.93	1.88
Model fit (BIC)	1514.5	1639.2	1336.6	1587.6				

*Longitudinal Mixed Model, n = 90 children, Raw scores of the State-Trait Operation Anxiety Inventory, BIC, Bayesian Information Criterion*,

* *p ≤ 0.05*,

** *p ≤ 0.01*,

*** *p ≤ 0.001*.

### Patient Satisfaction

The results of the patient satisfaction evaluation regarding the received information are shown in Table 4. In total, children and parents rated their satisfaction equally high but showed no significant group differences. In one item (Table 4, Item 4), both children and parents rated their reduction in worry with the received information as being significantly higher (children *p* = 0.01; parents *p* = 0.004) in the IG than in the CG (b).

**Table 4:** Subjective evaluation of intervention.

**Item**	**Self-report**					**Parent-report**				
	**CG (TAU)**		**IG (Video)**			**CG (TAU)**		**IG (Video)**		
	***M***	**SD**	***M***	**SD**	***p***	***M***	**SD**	***M***	**SD**	***p***
**Patient information**										
1. All questions answered	3.3	0.82	3.3	0.87	0.984	3.4	0.74	3.3	0.88	0.798
2. Easy to understand	3.25	0.87	3.50	0.86	0.209	3.38	1.00	3.73	0.45	0.073
3. Worries reduced	2.33	1.10	3.02	1.20	0.010^**^	2.78	1.07	3.46	0.73	0.004^**^
4. Uncertain	1.08	1.08	1.38	1.46	0.306	0.59	0.91	0.97	1.44	0.191
5. Has caused anxiety	0.79	0.85	1.00	1.15	0.394	0.72	1.20	0.28	0.65	0.070
6. Were correct	2.97	1.19	3.06	1.26	0.773	3.25	0.88	3.17	1.14	0.768
7. Overall satisfied	3.29	0.94	3.19	1.14	0.691	3.38	0.91	3.14	1.03	0.345
8. Total evaluation	8.11	1.33	8.14	1.96	0.933	8.16	1.75	8.68	1.36	0.188
**Video**										
9. Easy to understand			3.74	0.95				3.96	0.20	
10. Visually appealing			3.57	0.78				3.73	0.53	
11. Total satisfaction			3.43	0.98				3.58	0.58	
12. Total evaluation Video 1–10			8.44	2.21				9.04	1.18	

*Item 1 and 12 can be rated on a 1 to 10-point scale to evaluate the general satisfaction with the obtained patient information. Standardized items with a 5-point response scale derived from previous studies (21) (0 = strongly disagree, 1 = disagree, 2 = neither agree nor disagree, 3 = agree, 4 = strongly agree). The IG filled out four additional items for evaluating the quality and satisfaction of the video*.

** *p < = 0.01*.

## Discussion

The purpose of this study was to evaluate whether a child-friendly, educational video with a laying technique could reduce pre-operative anxiety in children undergoing elective surgery compared with the standard information procedure (CG). To our knowledge, this study also represents the first time that the STOA has been used to measure children's self-reported anxiety levels before elective surgery.

In contrast to previous research (1, 16, 23–26), no significant effects were found in the reduction of pre-operative anxiety among children following the addition of a video to TAU before surgery compared with TAU alone. Both the children and their parents reported their anxiety using a validated questionnaire (STOA). The measurement of state and trait anxiety at three different time points allowed us to comprehensively investigate the development of anxiety over the perioperative time. Although we were unable to identify significant differences between the groups, significant reductions in pre-operative state anxiety were observed at t2 compared to t1 for the children in both groups (IG and CG). The depicted course of anxiety levels shows coherence with a stressful situation, with higher state anxiety before the surgical intervention (7). Trait anxiety remained the same over the course of the three measurement time points, confirming the results reported by previous studies indicating that trait anxiety follows a constant course, representing a stable, consistent personality characteristic (1, 7). We failed to identify any decrease in the pre-operative state (and trait) anxiety in parents regarding their children's operations from t1 to t2. Fernandes et al. reported similar non-significant group differences (16). Interestingly, high anxiety scores in parents have been shown to predict their children's anxiety scores (5, 26–29).

Recent studies have used the modified Yale Pre-operative Anxiety Scale (mYPAS) as the gold standard for the measurement of observer-rated child anxiety, although some limitations associated with observer bias have been noted (30–32). Compared with this and other questionnaires that have been applied to measure anxiety, the STOA appears to represent a good alternative. It can measure the self-reported anxiety of both children and their parents, can be used to distinguish state from trait anxiety, and includes both cognitive and emotional subscales. With remark the STOA is a validated questionnaire used in a previous study only with adults (2).

In this study, the time point for the intervention was chosen as the day of surgery, which was also the timing used to provide the standard information procedure. However, the day of the operation, particularly the period shortly before the induction of anesthesia, is particularly stressful for the child (33). The existing anxiety level might strongly influence the ability of the children to cognitively process procedural information in a short period of time. This may affect the ability of this information to reduce anxiety levels in highly stressful situations (18). Other studies have indicated that significant reductions in anxiety could be achieved when presenting the pre-operative program at least 1 day prior to surgery (28, 34).

Child's age has been identified as an important variable in the prediction of the child's pre-operative anxiety level (19). In the present study, older children reported higher anxiety levels than younger children. Younger children might have felt more responsive to the video because of the simple format, the child-friendly language, and illustrations. Older children may have reported more anxiety because the information regarding the expected medical procedures was not age-appropriate and sufficiently detailed (14). Recent research has successfully used interventions based on video distraction, such as cartoons (30) and streaming video clips (31), during anesthesia induction. Among the younger children the video might have functioned more as a distraction rather than as a source of information and thereby explains the better response in children.

Boys reported lower anxiety values than girls in the present study, which agrees with prior results among children suggesting that gender is an important predictor of anxiety before surgery (16, 35).

This study used an informational education method, in which procedural information was mediated using a peer-modeling concept. Batuman et al., using mYPAS scores, showed significantly reduced anxiety scores in the IG compared with the CG when using a role-playing model. Peer-modeling videos can assist children to cope with an upcoming event through the observation of a child peer in a similar situation (1, 16, 23, 25).

The video did not provide detailed information regarding any surgical procedures, and the children may not have felt directly addressed because the video did not reflect their personal medical situation. Previously, others have stated that information should include procedural information regarding the events that will occur and sensory information regarding what children might expect, including the sensation of pain (20, 25).

Children and parents in the IG watched the educational video in the patient, holding, or examination room. The use of settings in which the children and their parents feel familiar and comfortable might impact the effectiveness of video interventions presented prior to hospitalization and surgery (36). Wakimizu et al. reported significant differences when an intervention group was presented with a pre-operative video and complementary booklet prior to hospitalization and additional at home compared with a control group that received the same pre-operative video only before hospitalization (36).

The standard care procedure already resulted in anxiety reduction, as reported by both children and parents, which may also account for the non-significant difference observed between the IG and the CG. Children's hospitals typically provide family-centered and age-appropriate information (37). The hospital staff is trained to communicate child-friendly information regarding the operation and the hospital stay and to involve parents in the operative setting. Personal contact between hospital staff and patients appears to be very effective in reducing children's anxiety (38). However, time and well-trained personal are not always freely available in clinical practice, and additional technology-based programs might be helpful for the implementation of standardized patient information (39).

In addition to objective measures of anxiety, a subjective evaluation regarding various aspects of the video was performed. Both, the children and their parents in the IG reported significant reductions in their worries compared with those in the CG. A short educational video may be a useful tool for informing children and parents regarding operational procedures and preparing them adequately for the upcoming surgery, as both children and their parents desire comprehensive information regarding perioperative procedures (10).

Important limitations of the present study should be considered. The information mediated by the educational video might have been insufficient due to the short time period before surgery. The content and format of the patient information used in this trial were not adapted to each child's age- and developmentally related concerns and fears. Furthermore, the questionnaire measuring child and parent satisfaction has not been validated in the study's target group. Three items have been selected from the original scale with regard to the study purposes.

Considering that the children answered the STOA at t3, it is possible that the children were influenced by medication like painkillers when answering the questions regarding their surgical anxiety and thus reported less anxiety. In addition, procedures for which hospital admission was required after the operation of the child could have influenced the post-operative anxiety and pain level of the child. As this was not the main focus of this study, we did not collect this data. Unfortunatly we didn't register the time of the medical counseling in both groups, which could have been an important factor in a busy unit.

The educational video could have been implemented as a supplementary and patient-orientated source of information in the pre-operative medical educations about anesthesia and surgery. A possible opportunity in our study and a possible outlook for further studies could be to provide a link so that children and parents could view the video at home or at a time of their choosing before surgery. In addition they would have the possibility to ask questions about the educational video and its content in the pre-operative medical information visit.

No pre-test has been performed to investigate the effects of an educational video on children with regard to pre-operative anxiety levels.

## Conclusion

Pre-operative anxiety is an important component of pediatric surgery. In this study we couldn't find an effect for the self-reported pre-operative anxiety with a video as an additional measure to the standard procedure. The optimization of well-chosen, pre-operative, educational programs remains necessary in terms of the timing before surgery, format, and content of the program. The STOA has been demonstrated to be a reliable and factorially valid measurement instrument for the parallel assessment of both state and trait anxiety among children and their parents. Further studies should include smaller age groups to test which types of media and intervention methods might be used to address the levels of anxiety experienced among children prior to surgery. Aside from influencing anxiety, audio-visual programs, available in a variety of languages could improve pediatric surgical care regardless of patient's origin and language barriers.

## Summary

This randomized controlled trial shows the effects of an educational video on the pre-operative anxiety of children and parents.

## Data Availability Statement

The original contributions presented in the study are included in the article/Supplementary Material, further inquiries can be directed to the corresponding author.

## Ethics Statement

The studies involving human participants were reviewed and approved by Ethics Committee of the Hamburg Medical Association (Number PV6045). Written informed consent to participate in this study was provided by the participants' legal guardian/next of kin.

## Author Contributions

VH conceptualized the study, conducted the data collection, analyzed the data, and drafted the initial manuscript. CB conceptualized and designed the study, analyzed the data, and reviewed and revised the manuscript. CW consulted on the statistical methods, analyzed the data, and approved the final manuscript. KR and MR reviewed and revised the manuscript. JT conceptualized and designed the study and the video and reviewed and revised the manuscript. All authors approved the final manuscript as submitted and agree to be accountable for all aspects of the work.

## Conflict of Interest

The authors declare that the research was conducted in the absence of any commercial or financial relationships that could be construed as a potential conflict of interest.

## Abbreviations

STOA: State-Trait Operation Anxiety Inventory
TAU: Treatment-as-usual
IG: Intervention group
CG: Control group
t1: timepoint 1 before intervention
t2: timepoint 2 after intervention
t3: timepoint 3 after surgery.

## Appendix

The 3-min child-friendly educational video explains the procedures in a hospital, when an injury happens, from the perspective of a young boy (peer-modeling). It shows how patients are getting prepared by the nursing staff and how they enter the operating room accompanied by a nurse and a parent. Furthermore, the video explains to whom patients can refer to if they need analgesic drugs during their hospital stay. The illustration appears on a flat and white screen with soft background music. This video uses a format so-called hand lay-style, where new scenes are wiped in or out with two hands. Videos like this visualize and simplify abstract and complex information in a short and simple way (40).

Video can be found under: https://www.uke.de/kliniken-institute/kliniken/kinderchirurgie/aufnahme/index.html
