# Supplementary material for: Effects of Educational Video on Pre-operative Anxiety in Children - A Randomized Controlled Trial
Source: Front Pediatr. 2021 May 12;9:640236. doi: 10.3389/fped.2021.640236 (PMC8149614; doi:10.3389/fped.2021.640236)
Supplement: Supplementary file 2 [file Data_Sheet_2.docx]

Date: __________ Time: __________ Number: __________

**Personal data:**

Age: _____

Gender: male □ female □

Mother tongue: _____________

Profession: _____________

| When I think about operations and anesthesia of my child **in general**, I worry that... | | | | | |  |
| --- | --- | --- | --- | --- | --- | --- |
| Nr. |  | Almost never  0 | Some-times  1 | Often  2 | Almost always  3 | |
| 1 | ...the hospital stay is very long. | □ | □ | □ | □ | |
| 2 | ...the anesthesia causes discomfort after the operation. | □ | □ | □ | □ | |
| 3 | ...the wound heals badly. | □ | □ | □ | □ | |
| 4 | ...there is pain after the operation. | □ | □ | □ | □ | |
| 5 | ...personal freedom is severely restricted. | □ | □ | □ | □ | |
| 6 | ...you have to suffer similarly to some of the other patients you see. | □ | □ | □ | □ | |
| 7 | ...you won't wake up from the anesthesia. | □ | □ | □ | □ | |
| 8 | ...further treatments are necessary after the operation. | □ | □ | □ | □ | |
| 9 | ...one is exposed to an impersonal apparatus. | □ | □ | □ | □ | |
| 10 | ...you do not know what it will be like when you wake up from the anesthesia. | □ | □ | □ | □ | |
| 11 | ...you can still feel something of the operation despite the anesthetic. | □ | □ | □ | □ | |
| 12 | ...the physical situation has become even worse after the operation. | □ | □ | □ | □ | |
| 13 | ...prolonged or chronic pain occurs. | □ | □ | □ | □ | |
| 14 | ...perhaps the decision to have the operation was not the right one. | □ | □ | □ | □ | |
| 15 | ...narcotic complications such as paralysis or circulation problems occur. | □ | □ | □ | □ | |
| 16 | ...hospital-associated infections occur. | □ | □ | □ | □ | |
| 17 | ...one feels disturbed by the sight of other suffering patients. | □ | □ | □ | □ | |
| 18 | ...complications such as fainting or bleeding occur after the operation. | □ | □ | □ | □ | |
| 19 | ...one is treated wrong. | □ | □ | □ | □ | |
| 20 | ...during anesthesia you are exposed to the doctors and have no control over yourself. | □ | □ | □ | □ | |

Please indicate how well the following statements describe your **current feelings**.

| Nr. |  | Not at all  0 | Some-what  1 | Mode-ratly so  2 | Very much so  3 |
| --- | --- | --- | --- | --- | --- |
| 21 | I feel tense inside. | □ | □ | □ | □ |
| 22 | I am excited. | □ | □ | □ | □ |
| 23 | I am nervous. | □ | □ | □ | □ |
| 24 | I feel uncomfortable. | □ | □ | □ | □ |
| 25 | I feel anxious. | □ | □ | □ | □ |
| 26 | I think about my situation. | □ | □ | □ | □ |
| 27 | I worry that something might go wrong. | □ | □ | □ | □ |
| 28 | I am worried. | □ | □ | □ | □ |
| 29 | I worry about my physical condition. | □ | □ | □ | □ |
| 30 | I am preoccupied. | □ | □ | □ | □ |

Thanks for your participation!
